# MiR-124 Suppresses Growth of Human Colorectal Cancer by Inhibiting STAT3

**DOI:** 10.1371/journal.pone.0070300

**Published:** 2013-08-05

**Authors:** Jufeng Zhang, Yanxin Lu, Xupeng Yue, Huiming Li, Xia Luo, Ying Wang, Kepeng Wang, Jun Wan

**Affiliations:** 1 School of Life Science, Guangdong Pharmaceutical University, Guangzhou, China; 2 Biomedical Research Institute, Shenzhen-PKU-HKUST Medical Center, Shenzhen, China; 3 Experimental Research Center, First People’s Hospital, School of Medicine, Shanghai Jiaotong University, Xuhui, Shanghai, China; 4 College of Engineering, South China Agricultural University, Guangzhou, China; 5 Division of Life Science, Hong Kong University of Science and Technology, Hong Kong, China; H.Lee Moffitt Cancer Center & Research Institute, United States of America

## Abstract

Emerging evidence indicate that microRNAs (miRNAs) may play important roles in cancer. Aberrant expression of miRNAs has been frequently identified in different human malignancies, including colorectal cancer (CRC). However, the mechanism by which deregulated miRNAs impact the development of CRC remains largely elusive. In this study, we show that miR-124 is significantly down-regulated in CRC compared to adjacent non-tumor colorectal tissues. MiR-124 suppresses the expression of STAT3 by directly binding to its 3′-untranslated region (3′-UTR). Overexpression of miR-124 led to increased apoptosis of CRC cells and reduced tumor growth in vitro and in vivo. Knocking down STAT3 expression by specific siRNA suppressed the growth of CRC cells in vitro and in vivo, resembling that of miR-124 overexpression. Moreover, overexpression of STAT3 in miR-124-transfected CRC cells effectively rescued the inhibition of cell proliferation caused by miR-124. These data suggest that miR-124 serves as a tumor suppressor by targeting STAT3, and call for the use of miR-124 as a potential therapeutic tool for CRC, where STAT3 is often hyper-activated.

## Introduction

Colorectal cancer (CRC) is the third leading cause of deaths among all human malignancies [1], [2]. Most CRCs arise sporadically, with sequential mutations in *APC/β-catennin, K-Ras, COX-2*, and *p53* signaling along the process of cancer initiation, progression and metastasis [3]–[5]. In addition to cancer cell-intrinsic mechanisms mediated by these oncogenes and tumor suppressor genes, interaction between cancer cells and other cells in tumor stroma also plays important roles in shaping the development of cancers [6]. In a process mimicking natural selection, cancer cells co-evolve with their microenvironment and are selected by their ability to remodel their surroundings for survival, proliferation, and metastasis to distant sites [6], [7].

*STAT3* is a critical link between tumor cells and their microenvironments by regulating both tumor growth and tumor-associated inflammation [8], [9]. In cancer cells, *STAT3* plays important roles in tumor growth and progression [10]. Activated *STAT3* can be detected in multiple human cancers, including those of colon, skin, gastric, breast, lung and others [10]–[13]. Presence of nuclear-localized *STAT3* in human cancers implicates that *STAT3* may serve as an oncogene to promote cancer development. Indeed, conditional deletion of *STAT3* in colonic epithelial cells and in hepatocytes resulted in reduced tumor development in mice [10], [14]. In a mouse model of AOM/DSS-induced colitis-associated CRC, deletion of *STAT3* in enterocytes resulted in reduced tumor load in the intestine [15], [16]. *STAT3*, activated by pro-inflammatory cytokines like IL-6 from tumor-infiltrating myeloid cells, promotes both survival and growth of transformed intestinal epithelial cells. Deletion of IL-6 or its cognate receptor gp130 in AOM/DSS model resulted in reduced tumor size and tumor number, whereas further potentiation of *STAT3* activity by introducing hyper-active gp130 led to increased tumor diameter and tumor count [15], [16]. *STAT3* regulates survival of tumor cells by activating genes that confer resistance against apoptosis. These *STAT3*-dependent anti-apoptotic genes include *Bcl-xL, Bcl-2, c-IAP2, Mcl-1* and *Survivin* [13], [15]–[17]. Deletion of *STAT3* in cancer cells resulted in reduced expression of these pro-survival genes and increased cancer cell apoptosis [10], [15], [16]. Additional *STAT3* targets include genes that promote cell proliferation, like *Cyclins B* and *D*, and *c-Myc* [9], [16], which explains the reduced tumor sizes upon *STAT3* ablation [15], [16]. By promoting tumor cell survival and growth, *STAT3* serves as a signal integrator to enable transformed cells to survive and proliferate in response to stimuli originated from stromal cells in tumor microenvironment [5].

MiRNAs are small non-coding RNAs of ∼22 nt that silence gene expression by suppressing translation of mRNA into proteins [18]–[20]. MiRNAs are estimated to regulate more than 60% of genes in mammals, making them a major party in cell behaviors [21]. MiRNAs are involved in multiple cellular functions including proliferation, apoptosis and differentiation, and are implemented in diverse physiological and pathological processes ranging from development to cancer [18], [19], [22], [23]. The role of miRNAs in cancers have been well-demonstrated [24]–[27]. MiRNAs can serve as either oncogenes or tumor-suppressor genes depending on the nature of their targets. The first evidence showing involvement of miRNA in cancer came from a study in chronic lymphocytic leukemia (CLL), where *miR-15a* and *miR-16-1* are frequently deleted [28]. Later studies further demonstrated tumor-suppressing roles of *miR-15a* and *miR-16-1* by identifying *BCL2* as their regulatory target, which is an anti-apoptotic gene that is often over-expressed in many types of human cancers [29]. Another example of tumor-suppressive miRNA is the *let-7* family that inhibits the expression of *Ras* oncogene [30]. *Let-7* family miRNAs are located in fragile regions of human genome, and their loss indicates poor prognosis in human cancers [31], [32].

Multiple miRNAs with aberrant expression have been identified in different human malignancies, including CRC [33]–[35]. Genes that are regulated by these cancer-associated miRNAs include *cox2*, *apc, K-Ras, egfr* and more, that are important for initiation and progression of CRC [34]. Among them, miR-124 is an interesting target to study in cancer development. There have been extensive studies on the role of miR-124 in the nervous system, where it regulates neuronal development and neural plasticity by targeting Notch signaling and other genes involved in neuron differentiation and activation [36]. MiR-124 is down-regulated in medulloblastoma and cervical cancer [37], [38], and is also involved in an inflammatory feedback loop in hepatocellular carcinoma (HCC) [39]. The role of miR-124 in CRC, and the mechanism by which miR-124 regulates cancer development, remain largely unknown.

In this study, we aimed to decipher the role of miR-124 in CRC development. We demonstrate that miR-124 is downregulated in human CRC. MiR-124 targets the 3′ untranslated region (3′UTR) of STAT3 to suppress its expression. By downregulating STAT3, miR-124 induces programmed cell death in human CRC cells and suppresses the growth of CRC tumors in vivo.

## Materials and Methods

### Cell Culture and Reagents

SW480 and LoVo human CRC cell lines were obtained from the Cells Bank of the Chinese Academy of Science (Shanghai, China). All cells were grown in DMEM supplemented with 10% fetal bovine serum (FBS), 2 mM glutamine, 100 U/ml penicillin and 100 µg/ml streptomycin. All cells were incubated at 37°C in a humidified chamber supplemented with 5% CO_2_.

*MiR-124* precursor (*Pre-miR-124*), miRNA precursor control (*Pre-Scrambled miRNA*), antisense *miR-124* oligonucleotide (*AS-miR-124*), CY3-labled *miR-Scramble*, antisense miRNA control (*AS-Scrambled* miRNA), siRNA against *STAT3* (*STAT3-siRNA*), and scrambled siRNA-oligonucleotide (*siRNA-control*) were purchased from Ambion (Austin, TX, USA). *STAT3* antibody was purchased from Cell Signaling Technology (Danvers, MA, USA). *PcDNA3.0-STAT3*, a STAT3 expression vector was constructed by our laboratory.

### Patient Specimens and RNA Extraction

Human CRC samples were obtained from 90 surgical patients in the department of gastroenterology, Peking University Shenzhen Hospital. Adjacent normal mucosa samples located at least 2 cm from the margins of the tumor (polyp or carcinoma) were used as controls. All tumors were adenocarcinomas whereas mucinous carcinomas (when more than 50% of the tumor volume was composed of mucin) were excluded. Colorectal cancers were staged according to the Dukes classification system: Dukes A (T1–T2, N0, and M0; n = 26), Dukes B (T3–T4, N0, and M0; n = 16), Dukes C (any T, N1–2, M0; n = 38) and Dukes D (any T and any N and M1; n = 10). CRC samples were collected from patients undergoing bowel resection. Collected samples were stored in liquid nitrogen. All patients were informed about the aims of the specimen collection and gave written consent in accordance with the ethical guidelines of Peking University. The study was approved by the ethical committee of Peking University Shenzhen Hospital. MiRNA was extracted from fresh tissues using the Ambion mirVana miRNA isolation kit (Ambion) according to the manufacturer’s instructions.

### Detection of Mature *miR-124* by TaqMan Real-time RT-PCR

Real-time RT-PCR analysis for mature *miR-124* was carried out in triplicate using TaqMan MicroRNA assays kit (Ambion) according to manufacturer’s instruction. RT reaction contained 10 ng total RNA, 1 mM dNTPs, 50U Multiscribe Reverse Transcriptase, 1.5 µl 10×RT buffer, 0.188 µl RNase inhibitor, and 3 µl 5×TaqMan MicroRNA RT primer in each reaction (15 µl). The RT reaction was conducted under the following conditions: 16°C for 30 min; 42°C for 30 min; 85°C for 5 min; and then held on 4°C. After the RT reaction, the cDNA products from RT reaction were diluted 15 times. PCR was carried out with 1.33 µl of the diluted products in 20 µl PCR reaction containing 1 µl of TaqMan MicroRNA Assay and 10 µl of TaqMan Universal PCR Master Mix. Amplification was performed as follows: 95°C for 10 min, followed by 40 cycles of 95°C for 15 s and 60°C for 60 s. Relative expression was calculated using the comparative CT method and normalized to the expression of *RNU6B* (Ambion).

### Quantitative Real-time RT-PCR Analysis of *STAT3* mRNA Expression

Total RNA was extracted from cell lines transfected with *Pre-miR-124* or control by TRIzol reagent (Invitrogen, Carlsbad, CA, USA). cDNA was synthesized with reverse transcriptase M-MLV kit (TaKaRa, Dalian, China) using 1 µg total RNA and 50 µM Oligo (dT) primer in 10 µl reaction volume. Reverse transcription was performed under the following program: 30 min at 70°C, then cooled on ice immediately, 1 hour at 42°C, 15 min at 70°C and then held at 4°C. RT products were diluted 10-fold. Real-time PCR was done in triplicates with iQ-SYBR Green Supermix (Bio-rad, CA, USA) and Icycler Instrument (Bio-rad, CA, USA) using 1 µl diluted cDNA as template in a 20 µl reaction volume. PCR reaction was carried out as following: 95°C for 3 min and 40 cycles of 95°C for 20 s, 55°C for 30 s, and 72°C for 20 s. The primers used for real-time PCR are: *STAT3* forward primer: 5′-ATCACGCCTTCTACAGACTGC-3′, Reverse primer: 5′-CATCCTGGAGATTC.

TCTACCACT-3′. *GAPDH* forward primer: 5′-CCACTCCTCCACCTTTGAC-3′, Reverse primer: 5′-ACCCTGTTGCTGTAGCCA-3′. Relative expression was calculated by 2^–ΔΔCt^ method.

### Transfection

For RNA oligonucleotides, cells were transfected with siPORT NeoFX (Ambion) with 50 nM siRNA or 100 nM miRNA. For plasmid, cells were transfected with 4 µg DNA in 35 mm wells by Lipofectamine 2000 (Invitrogen). In the rescue experiment, cells were co-transfected with 100 nM miRNA and 3 µg plasmid in 35 mm wells. Transfection efficiency was estimated by CY3-labeled miR-Scramble for RNA oligonucleotides or by GFP-expressing vector for plasmid.

### Proteomic Analysis

SW480 cells were transfected with *Pre-miR-124* or *Pre-Scrambled* miRNA control. 48 h after transfection, cell protein were extracted and protein concentration was determined using Bio-Rad protein Assay (Bio-Rad, CA, USA). Proteins were used for 2-DGE as described by Bio-Rad manual. Mass spectrometry analysis was performed at the Teaching Center of Biology Experiment, School of Life Sciences, Sun Yat-Sen University (Guangzhou, China). 2-DGE with the first dimension isoelectrofocusing was carried out pH3-10 IPG ready strips, and in the second dimension was separated by 8–14% gradient SDS-PAGE.

### Plasmid Construction

We amplified the 3′-UTR segment of *STAT3* containing predicted *miR-124* target site by PCR from SW480 cell genomic DNA, and inserted it into the SpeI/HindIII sites downstream the luciferase gene in pMIR–REPORT Luciferase miRNA Expression Reporter Vector (Ambion). According to the gene bank sequence of *STAT3*, we designed the following primers to amplify the 3′-UTR of *STAT3* by PCR: Forward primer, 5′-CGGACTAGT AAATGAGTGAATGTGGGTG-3′, and reverse primer, 5′-CCAAGCTTTGTTGCTGGAGAAGTAAGAG-3′. 3′-UTR segment of *STAT3* with mutant *miR-124* target site was generated by overlap-PCR using wild type 3′-UTR construct as template. The following primers were used to generate 3′-UTR containing mutant *miR-124* target site: 3′-UTR-*STAT3*-M-reverse, 5′-CCAGCCCTGAGGACTACACCACAGAAACAACCTAGCC-3′,3′-UTR-*STAT3*-M-forward, 5′-GTTTCTGTGGTGTAGTCCTCAGGGCTGGGATACTTCTG-3′. All the positive constructs were identified by restriction digestion and confirmed by DNA sequencing.

### Luciferase Assays

SW480 cells were transfected with luciferase constructs containing 3′-UTR of *STAT3* (with the wild type or mutant *miR-124* target sites) and/or *Pre-miR-124* or *Pre-Scrambled* miRNA control. 48 h after transfection, cells were harvested for luciferase assays using a luciferase assay kit (Promega, Madison, WI, USA) according to manufacturer’s protocol. *pMIR-REPORT-β-gal* was used for normalization.

### Cell Proliferation Assay

SW480 and LoVo cells were transfected with *Pre-miR-124* or *Pre-Scrambled miRNA* control as described above. 6 hours after transfection, cells were counted and plated at a density of 3×10^3^ cells per well in 96-well plates and 3×10^5^ cells per 30 mm-plate in triplicates. WST-1 assays were performed at 72 hours post-transfection. For WST-1 assay, spectrophotometry was performed at λ = 450 nm and λ ref = 690 nm after incubation with 10 ul WST-1 (Roche, New York, NY, USA) for 3 hours.

### Immunofluorescence Assay

SW480 and LoVo cells were transfected with *Pre-miR-124* or *Pre-Scrambled miRNA* control. 48 h after transfection, cells were fixed with 4% paraformaldehyde and permeabilized with 0.5% Triton X-100 in PBS. Rabbit antibodies against *STAT3* (Cell Signaling Technology, BSN, USA) was used as primary antibody, and FITC-conjugated goat anti-rabbit IgG (Chemicon International, Temecula, CA, USA) was used as secondary antibody to visualize *STAT3*. Nuclei were stained with DAPI.

### Western Blotting

Total protein was isolated from cell lines transfected with RNA oligonucleotide and/or plasmid DNA in cell lysis buffer (50 mM Tris-HCl, pH 7.5, 150 mM NaCl, 1% Triton X-100, 1 mM EDTA, 1 mM PMSF and 1% sodium deoxycholate). Protein concentration was measured using Bio-Rad protein assay kit. Protein samples were separated by 10% SDS polyacrylamide gel and transferred to a polyvinylidene difluoride membrane (Amersham, Buckinghamshire, UK). The blots were probed with antibodies against *STAT3* and phospho-*STAT3* (Cell Signaling Technology, BSN, USA), followed by a secondary horseradish peroxidase-conjugated antibody. The antigen-antibody complexes were visualized using the enhanced chemiluminescence kit (Roche) as recommended by the manufacturer.

### Apoptosis Analysis

SW480 and LoVo cells were transfected with *Pre-miR-124* or *Pre-Scrambled miRNA* control. After transfection 72 h, cells were stained with propidium iodide and anti-annexin-V antibody, and analyzed by flow cytometry.

### Animal Experiments

Specific pathogen-free female athymic BALB/c nude mice, 4–6 weeks old (20–30 g), were obtained from the Guangdong medical laboratory animal centre. Mice were housed 5 per cage and allowed free access to food and water. This study was carried out in strict accordance with the recommendations in the Guide for the Care and Use of Laboratory Animals of the National Institutes of Health. The protocol was approved by the Committee on the Ethics of Animal Experiments of the Shenzhen-PKU-HKUST Medical Center (Permit Number:158). All surgery was performed under sodium pentobarbital anesthesia, and all efforts were made to minimize suffering. SW480 cell lines were transfected in vitro with RNA oligonucleotide and/or plasmid (pcDNA3.0-STAT3) vector. 24 hours after transfection, 10^7^ viable cells were suspended in 100 µl PBS and subcutaneously injected into right dorsal lumbar region of nude mice. At least 7 mice were employed per group to test the effect of miR-124 in tumor growth. Tumor growth was assessed by measuring bidimensional diameters twice a week with calipers. The tumor volumes (V) were calculated using the following formula: V = A×B^2^/2, where A represents the larger diameter and B is the smaller diameter. 40 days after cell injection, mice were sacrificed for tissue analysis.

### Immunohistochemical Analysis and TUNEL Assay

Transplanted tumors were resected from host mice, fixed in 10% formalin, paraffin embedded, and cut into 4 µm thick sections. Sections were deparaffinized, rehydrated in xylene followed by graded alcohols, and microwave antigen retrieved with 10 mM citrate buffer solution (pH 6.0 for 15 minutes). After inactivation by exposure to 3% H_2_O_2_ for 10 min to block the endogenous peroxidase, sections were incubated with 10% goat serum in PBS for 30 minutes to block non-specific antibody binding. Sections were incubated with STAT3 antibody (Cell Signaling, #9132) at 1∶200 dilution overnight at 4°C and then washed in PBS. Secondary antibody (Biotinylated anti-rabbit IgG, dilution 1∶200) was applied and sections were incubated for 30 minutes at room temperature. After washing with PBS, sections were incubated with peroxidase-labelled streptavidin complex for 20 minutes at room temperature. Sections were then incubated with a solution of 3% diamino-benzidine (DAB) as the chromogen for 20 minutes. Fromowitz’s standard was used to semiquantitatively assess the staining of STAT3 [37].

TUNEL staining was performed on 6-µm sections of the excised tumors. Sections were deparaffinized prior to the labeling reaction. TUNEL assay was carried out using the TumorTACS In Situ Apoptosis Detection Kit (Trevigen Inc. USA). This assay specifically detects apoptotic cells when examined through the Zeiss microscope.

### Statistical Analysis

Significance analysis of microarrays (SAM) was used to identify miRNAs differentially expressed between samples (FDR = 0). *MiR-124* expressions in CRC tumors and adjacent non-tumor tissues were compared by the Mann-Whitney U test, and in the evaluation between early and advanced stage tumors. A comparison of means among two or more groups was performed using one-way analysis of variance or the Student’s t-test. All numerical data were expressed as mean ± SD. P-value less than 0.05 was considered significant. Statistical analyses were performed using Graphpad Prism 5.0 (GraphPad Software, San Diego, CA) and SPSS software (version 11).

## Results

### *MiR-124* is Down-regulated in Human CRC

**Figure 1:**
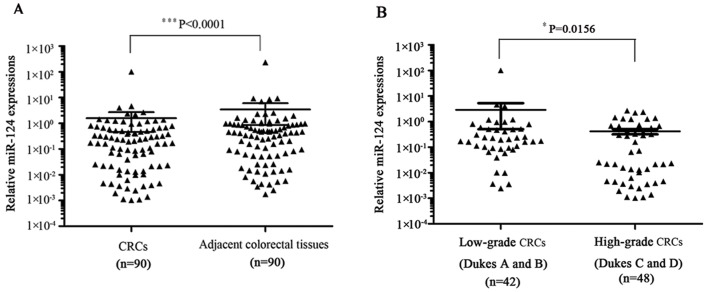
*MiR-124* is down-regulated in human CRC. (A) qRT-PCR analysis on the expression of miR-124 in CRC compared to adjacent nonmalignant colorectal tissues from 90 patients (P<0.0001). PCR reactions were performed in triplicates with RNU6B as internal control. (B) Association of miR-124 expression level with CRC progression (P = 0.0156).

To examine the expression profile of *miR-124* in CRC, we performed quantitative real-time RT-PCR using TaqMan assay in 90 paired tumor and normal colorectal specimens. As shown in Fig. 1A, we found significantly decreased *miR-124* in CRC samples (P<0.0001). Compared to colorectal cancer tissues from patients with low-grade CRC (Dukes A and B), high-grade (Dukes C and D) CRC tissues express even lower *miR-124* (P = 0.0156; Fig. 1B). These results suggest that *miR-124* may be involved in the pathogenesis of CRC.

### *STAT3* is a Target of Posttranscriptional Repression by *miR-124*

In order to identify targets of miR-124 we performed differential proteomic analysis from the protein of SW480 cells after treatment with either the Pre-miR-124 or Pre-Scrambled miRNA. After separating the proteins by isoelectric focusing and SDS-PAGE, we picked 12 protein spots that were down-regulated more than 2-fold in the cells treated with *Pre-miR-124* and then identified with mass spectrometry (Fig. S1A). Among them, *STAT3* has been implicated in tumorigenesis. *STAT3* is an important transcription factor that regulates diverse physiological and pathological processes including cancer. Knocking out *STAT3* in colon epithelial cells led to reduced tumor formation in a model of colitis-associated CRC in mice [15]. To test if STAT3 is a direct target of miR-124 regulation, we amplified 3′-UTR region of *STAT3* by PCR from SW480 genomic DNA and inserted it to the downstream of the luciferase reporter gene of *pMIR- REPORT* vector for luciferase assay, with *pMIR- REPORT β-gal* vector as control (Fig. S1Bi). Analysis with TargetScan software revealed a potential binding site for *miR-124* within the 3′UTR of *STAT3* (Fig. S1Biii). To test whether *miR-124* binds to 3′-UTR of *STAT3* and regulates *STAT3* expression through this site, we also constructed a luciferase vector fused to *STAT3* 3′UTR harboring a mutant *miR-124* response element (Fig. S1Bii). We then transfected these constructs into SW480 cells together with *Pre-miR-124* or *pre-Scrambled miRNA* and measured luciferase activity. As shown in Fig. S1C, transfection of *Pre-miR-124*, but not scrambled miRNA, significantly decreased luciferase activity of the luciferase reporter carrying WT *STAT3* 3′UTR. In contrast, neither *Pre-miR-124* nor *Pre-Scrambled miRNA* had any effect on the luciferase activity of the luciferase reporter containing mutant *miR-124* binding site. These data indicate that the *miR-124* directly interacts with the 3′UTR of *STAT3* and inhibits its expression.

**Figure 2:**
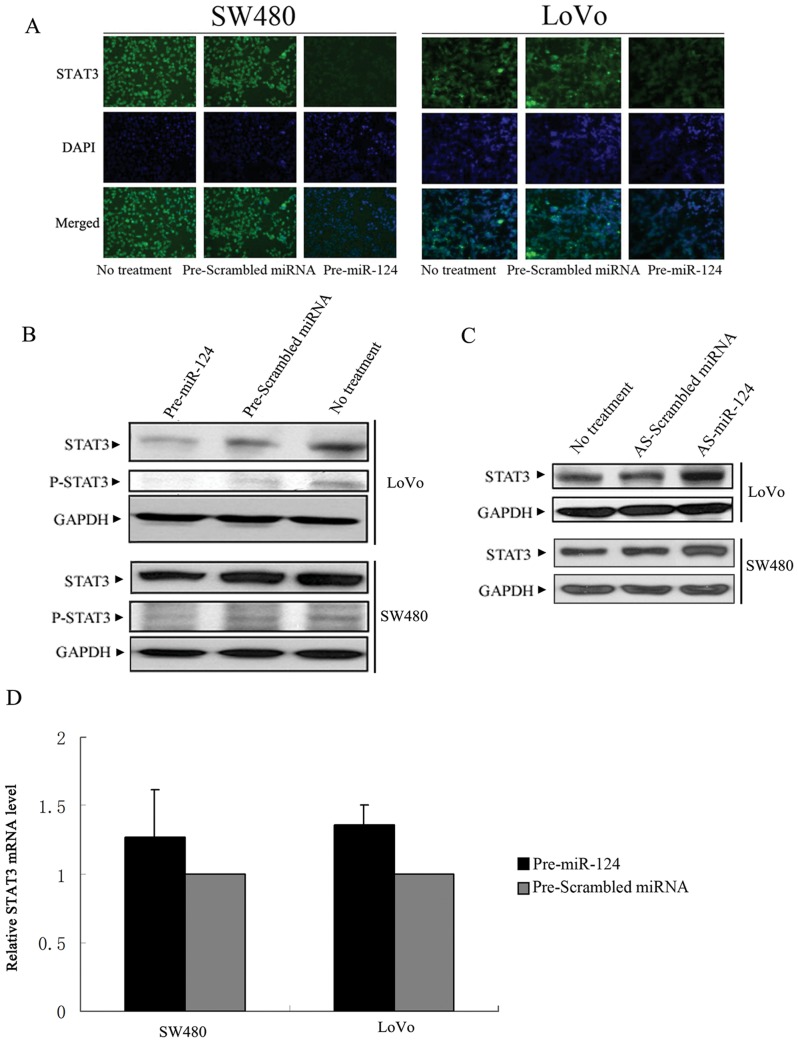
MiR-124 suppresses the expression of STAT3 in human CRC cells. (A–B) Effect of *miR-124* overexpression on endogenous *STAT3* level in human CRC cells. (A) SW480 and LoVo cells were transfected with *Pre-miR-124* and stained for *STAT3* by immunofluorescence analysis. Green, *STAT3* protein was immunostained with anti-*STAT3*; blue, nuclei were stained with DAPI. (B) SW480 and LoVo cells were transfected with *Pre-miR-124* and *STAT3* protein levels were examined by Western blotting analysis. (C) SW480 and LoVo cells were transfected with AS-miR-124. *STAT3* protein levels were examined by Western blotting. (D) The levels of *STAT3* mRNA were quantified by qRT-PCR analysis after transfection of Pre-miR-124. *GAPDH* were used as an internal control. Data are represented as mean±SD±SD.

We also investigated the effect of overexpressing *miR-124* on endogenous *STAT3* expression in human CRC cells. We used LoVo and SW480 cells with high levels of *STAT3* expression to conduct this experiment. Compared to cells untransfected or transfected with *Pre-Scrambled miRNA*, *Pre-miR-124*-transfected human CRC cells showed reduced *STAT3* expression evidenced by both immunostaining and Western blotting (Fig. 2A, B). Importantly, the level of active (Tyrosine-phosphorylated) *STAT3* was also reduced in *Pre-miR-124* -transfected CRC cells but not in control (Fig. 2B). To further validate the effect of *miR-124* on *STAT3* in human CRC cells, we neutralized endogenously expressed *miR-124* using antisense oligonucleotide (*AS-miR-124*). Following *miR-124* silencing, *STAT3* protein level was upregulated (Fig. 2C). In sum, these results showed that *STAT3* is a direct target of *miR-124*.

MiRNAs down-regulate their target genes by degradation of target mRNA or inhibition of mRNA translation. To investigate the mechanism of *STAT3* inhibition by *miR-124*, we tested the impact of *Pre-miR-124* transfection on *STAT3* mRNA stability. There was no difference in the levels of *STAT3* mRNA between cells transfected with *Pre-miR-124* and *Pre-Scrambled miRNA*, suggesting that miR-124 down-regulate *STAT3* expression by means of inhibiting translation (Fig. 2D).

### *MiR-124* Inhibits Growth of CRC

**Figure 3:**
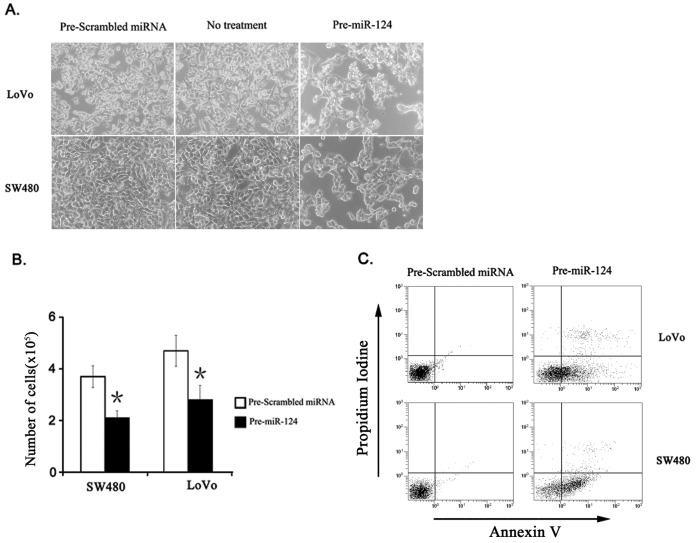
Overexpression of *miR-124* increases cell apoptosis in vitro. (A) SW480 and LoVo cells were transfected with *Pre-miR-124* or *Pre-Scrambled* miRNA and examined by light microscope. (B) Cells were transfected with *Pre-miR-124* or *Pre-Scrambled* miRNA, and cell growth was determined by WST1 assay. Columns represent mean of three independent tests. Bars, SD. *, P<0.05. (C) Cell apoptosis was measured by Annexin V and propidium iodine staining.

Knowing that *miR-124* is significantly downregulated in CRC, we investigated whether *miR-124* may serve as a tumor suppressor in CRC. To explore the effects of *miR-124* on cancer cell survival, we transfected *miR-124* precursor to human CRC cell lines SW480 and LoVo, and measured rate of proliferation and apoptosis. We confirmed transfection efficiency (>90% SW480 and LoVo cells) using CY3-labeled miR-Scramble (Ambion, data not shown). Overexpression of *miR-124* induced cell death evidenced by rounding up morphology of cells (Fig. 3A). Growth of both cancer cell lines was significantly inhibited at 48 hour following transfection of *Pre-miR-124* (Fig. 3B). Induction of apoptosis following overexpression of *miR-124* was confirmed by flow cytometry (FCM) (Fig. 3C).

**Figure 4:**
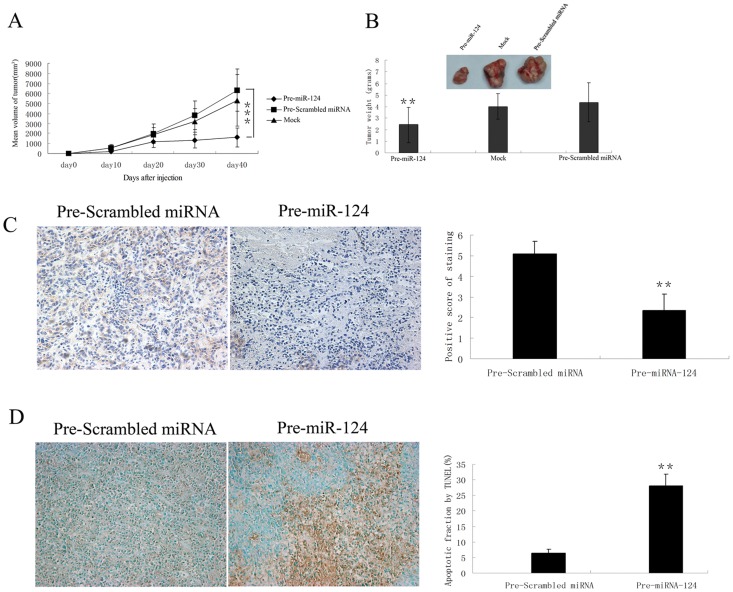
*MiR-124* inhibits tumor growth in vivo. (A) SW480 cells were transfected with *Pre-miR-124* or *Pre-Scrambled* miRNA. 24 hours after transfection, 10^7^ viable cells were suspended in 100 µl PBS and subcutaneously injected into the right dorsal lumbar region of nude mice. Tumor growth was assessed by measuring bidimensional diameters twice a week with calipers. (B) Tumor weight at the end of the study. On day 40 post treatment, all animals were sacrificed and tumors were removed and weighted. The data represent mean ± SD from at least 7 animals per group. (C) *STAT3* level is downregulated in *Pre-miR-124-*treated colorectal tumor tissues. Left: representative colorectal tumor tissue sections stained with antibody against *STAT3*. Right: *STAT3* staining intensity was graded by a pathologist and analyzed by Fromowitz’s standard. (D) Left: tumor sections were stained by TUNEL assay for apoptotic cells. Right: quantification of number of apoptotic cells. Columns, mean of three independent tests. Bars, SD, **, P<0.01, ***, P<0.001.

To further determine the role of *miR-124* in regulating tumor growth in vivo, we established xenograft model by injecting SW480 cells subcutaneously into nude mice. Prior to injection, SW480 cells were transfected with *Pre-miR-124* or *Pre-Scrambled miRNA*. Expression of *miR-124* following transfection was confirmed by TaqMan RT-PCR (data not shown). We found that 100% of mice injected with SW480 cells transfected with *Pre-Scrambled miRNA* developed measurable tumors after 10 days of cell inoculation. In contrast, tumor was not visible in two mice injected with SW480 cells treated with *Pre-miR-124*. Tumor growth was assessed by measuring bi-dimensional diameters twice a week with calipers. As shown in Fig. 4A, tumors originated from cells treated with *Pre-miR-124* were significantly smaller than those treated with *Pre-Scrambled miRNA*, or mock transfected (P<0.01) at day 40. Average tumor weight for *Pre-Scrambled miRNA* treated and untreated groups was 4.36±1.69 g and 3.99±1.11 g respectively, while in mice inoculated with *Pre-miR-124* treated cells it was 2.42±1.55 g (p<0.01) (Fig. 4B). *STAT3* expression level was also reduced in *Pre-miR-124*-transfected cell-derived tumors compared to control (Fig. 4C), suggesting the inhibition of *STAT3* by *Pre-miR-124* is conserved under physiological condition. Consistent with its role in promoting cell death in vitro, *Pre-miR-124*-transfected cell-derived tumor also showed dramatically increased cell death, which explains the reduced tumor size after transplantation (Fig. 4D).

Taken together, these results demonstrate that *miR-124* is a tumor suppressor in CRC.

### *MiR-124* Serves as a Tumor Suppressor by Targeting *STAT3*

**Figure 5:**
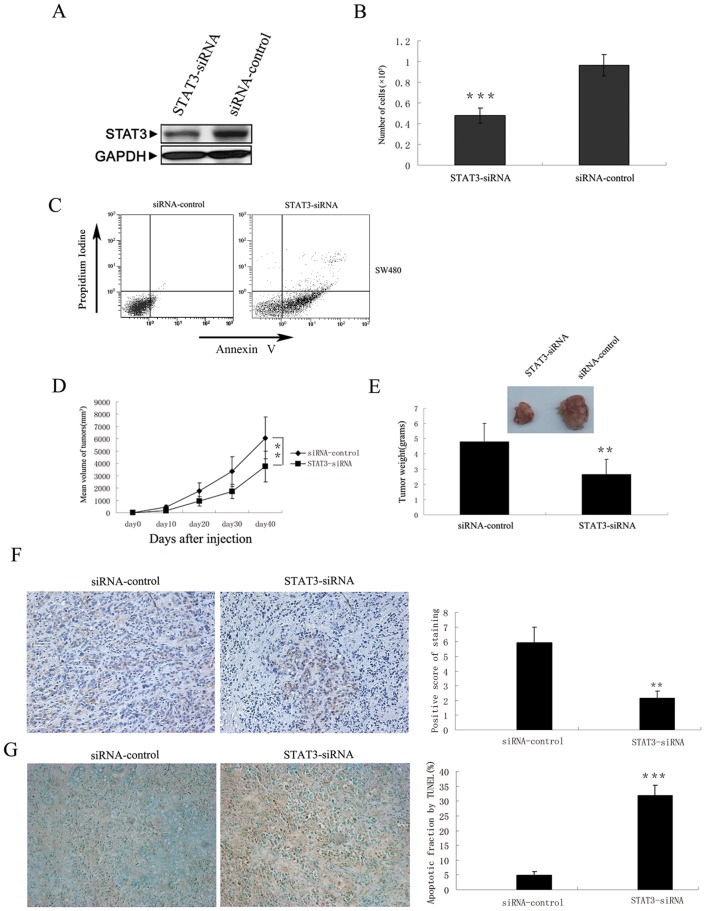
Knockdown of *STAT3* using *STAT3-siRNA* recapitulated tumour inhibition effect of *miR-124*. (A) Suppression of *STAT3* by *STAT3-siRNA* in SW480 cells. STAT3 protein levels were examined by Western blotting analysis. (B) SW480 cells were transfected with *STAT3-* or control-*siRNA* and cell growth was determined by WST1 assay. (C) Cell apoptosis was measured by Annexin V and propidium iodine staining. (D) SW480 cells were transfected with *STAT3-* or control-*siRNA*. 24 hours after transfection, 10^7^ viable cells were suspended in 100 µl PBS and subcutaneously injected into the right dorsal lumbar region of nude mice. Tumor growth was assessed by measuring bidimensional diameters twice a week with calipers. (E) Tumor weight at the end of the study. On day 40 post treatment, all animals were sacrificed and tumors were removed and weighted. Data represent mean ± SD from at least 7 animals per group. (F) *STAT3* level is downregulated in *STAT3-siRNA*-treated colorectal tumor tissues. Left: representative colorectal tumor tissue sections stained with antibody against *STAT3*. Right: *STAT3* staining intensity was graded by a pathologist and analyzed by Fromowitz’s standard. (G) Left: tumor sections were stained by TUNEL assay for apoptotic cells. Right: quantification of cell apoptosis comparing STAT3-siRNA-treated and control groups. Columns, mean of three separate experiments; Bars, SD, **, P<0.01, ***, P<0.001.

MiRNAs identify their targets through degenerate matching with target sequences. As a result each miRNA may inhibit expression of multiple genes. To ascertain that *STAT3* is indeed the downstream mediator of *miR-124*’s inhibitory effect on cancer cell survival and in vivo tumor growth, we designed siRNA against *STAT3* to specifically down-regulate *STAT3* expression and seek to recapitulate the effect of *miR-124* on CRC development. Transfection of *STAT3* siRNA (*STAT3-siRNA*) resulted in reduced *STAT3* expression in human CRC cells in vitro and in vivo (Fig. 5A, F). As expected, knocking down of *STAT3* by siRNA resulted in reduction of cells number (Fig. 5B). Compared to control cells, *STAT3-siRNA*-transfected human CRC cells also showed increased apoptosis in culture (Fig. 5C) and when transplanted in vivo (Fig. 5G). This reduced survival of cancer cells similarly led to reduced tumor size when transplanted into nude mice (Fig. 5D, E).

**Figure 6:**
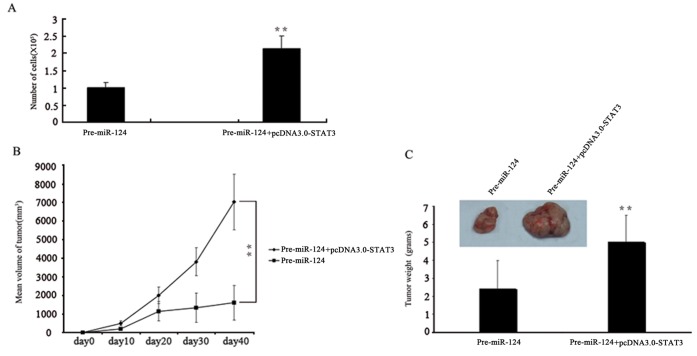
*STAT3* overexpression bypasses the tumor inhibition function of *miR-124*. (A) SW480 cells were transfected with *Pre-miR-124* and/or *STAT3* expression vector, and cell growth was determined by WST1 assay. (B) *STAT3* overexpression promotes growth of colon cancer cells transfected with *Pre-miR-124* in nude mice. (C) Tumor weight at the end of the study. Columns, mean of three independent experiments; Bars, SD, **, P<0.01.

We then performed rescue experiments to further validate that *STAT3* targeting is involved in *miR-124*–mediated antitumor properties in CRC cells. *STAT3* expression vectors, *pcDNA3.0-STAT3* was used to restore *STAT3* expression. Inhibition in cell growth by *miR-124*–overexpression was significantly attenuated by re-introduction of *STAT3* (Fig. 6A). Importantly, over-expression of *STAT3* in *miR-124*-transfected CRC cells completely rescues the reduction in tumor size when growing in vivo (Fig. 6B, C). These and previously described experiments show that the downregulation of *STAT3* by *miR-124* is an authentic mechanism of *miR-124*–mediated inhibition of tumor growth in CRC.

In summary, our study demonstrates that *miR-124* serves as a tumor suppressor by inhibiting translation of *STAT3* mRNA. Inhibition of *STAT3* by *miR-124* leads to increased cell apoptosis both in vitro and in vivo, and contributes to reduced tumor growth from transplanted human CRC cells.

## Discussion

The role of *miR-124* in CRC was not reported before. *MiR-124* was first demonstrated to be a “brain-specific” miRNA, and was shown to regulate Cocaine-induced neuronal plasticity by inhibiting the expression of *BDNF* [36]. *MiR-124* is frequently down-regulated in medulloblastoma, indicating its potential role in cancer [37]. Targets of *miR-124* in medulloblastoma include *SLC16A1*, which regulates lactic acid export during aerobic glycolysis [37]. *MiR-124* also inhibits the expression of *SNAI2* in human glioma [40]. Loss of *miR-124* in glioma cells enhances stem-cell like traits and increases invasiveness of these cells in vitro and in vivo [40]. *MiR-124* is also down-regulated in cervical cancers, but its target is not clear [38]. In mouse models of hepatocellular carcinoma, miR-124 is involved in an inflammatory feedback loop where it suppresses the expression of IL-6R and reduces STAT3 activation in transformed cells [39]. Transient inhibition of HNF4α initiates transformation of hepatocytes and downregulates miR-124, resulting in elevated IL-6/STAT3 signaling that promotes HCC development by further suppressing HNF4α through miR-24 and miR-629 [39]. In this article we show that miR-124 is down-regulated in human CRC. MiR-124 suppresses the survival of human CRC cells by inhibiting the expression of STAT3. Introducing miR-124 back to human CRC cells resulted in increased cell apoptosis in vitro and decreased tumor growth in vivo.

Aberrant expression of miRNAs can arise from a number of mechanisms, including deletion in fragile regions of genome where cancer-suppressing miRNAs locate, inherent or spontaneous mutations in miRNA genes, or methylation in the promoters of miRNAs [26], [41]–[45]. In human cervical cancers, miR-124 locus is frequently methylated, which contributes to its down-regulation [38]. The mechanism by which miR-124 is down-regulated in human CRC remains elusive.

STAT3 is activated in multiple human cancers and was shown to function as an oncogene [2]. Activation of STAT3 comes from phosphylation of its tyrosine-705 residue, dimerization, and nuclear translocation, followed by activation of its target genes [46]. Here we demonstrate that the expression level of STAT3 is also important in the settings of human cancers. STAT3 is known to promote development of CRC by inducing proliferation and survival of CRC-initiating cells [15], [16], [47]. We found that down-regulation of STAT3 by miR-124 leads to increased cancer cell death and reduced tumor load when transplanted in mice. MiR-124 therefore serves as a tumor suppressor through inhibition of STAT3 signaling, which explains its down-regulation in human CRC and other cancers. Of note, hyper-activation of STAT3 in human CRC also indicates poor prognosis [48], [49]. Given the link between miR-124 and STAT3, it would be interesting to test the potential of using miR-124 as diagnostic marker or therapeutic tool for human CRC.

In conclusion, our study demonstrates that miR-124 is dramatically down-regulated in human CRCs. MiR-124 promotes apoptosis of CRC cells by suppressing the expression of STAT3. Increased cell death resulted from reconstitution of miR-124 led to reduced tumor growth in immune-compromised mice. Our data indicated a novel role of miR-124 in CRC, and demonstrated potential to use miR-124 as diagnostic marker or therapeutic tool for human CRC.

## Supporting Information

Figure S1 ***STAT3*** **is a direct target of** ***miR-124*** **.** (A) Two dimensional gel electrophoresis analysis on whole cell extracts from SW480 cells treated with Pre-miR-124 or Pre-Scrambled control miRNA. Proteins in the gel were stained with CBB (Coomassie brilliant blue) G-250. 12 protein spots circled are presumably down-regulated by miR-124. Arrowhead represents STAT3. (B) Design of luciferase reporter vectors containing a CMV promoter driving expression of a luciferase cDNA fused to the *STAT3* 3′UTR (B.i) or to the mutated *STAT3* 3′UTR (B.ii). The *miR-124* WT binding site and mutated binding site in the 3′UTR of *STAT3* are shown in B.iii. (C) *MiR-124* binding site within *STAT3* 3′UTR mediates *miR-124* control of *STAT3* translation. SW480 cells were co-transfected with the luciferase constructs, *Pre-miR-124* or *Pre-Scrambled* miRNA control, respectively. Cell lysate was collected and assayed for luciferase activities 48 h after transfection. Cells were transfected with a *pMIR-REPORT* miRNA expression reporter as control. *Pre-miR-124* significantly decreases luciferase activity containing a WT *miR-124* binding site but not a mutant binding site.
(TIF)
