# Supplementary figures and images for: MiR-124 Suppresses Growth of Human Colorectal Cancer by Inhibiting STAT3
Source: PLoS One. 2013 Aug 5;8(8):e70300. doi: 10.1371/journal.pone.0070300 (PMC3734178; doi:10.1371/journal.pone.0070300)

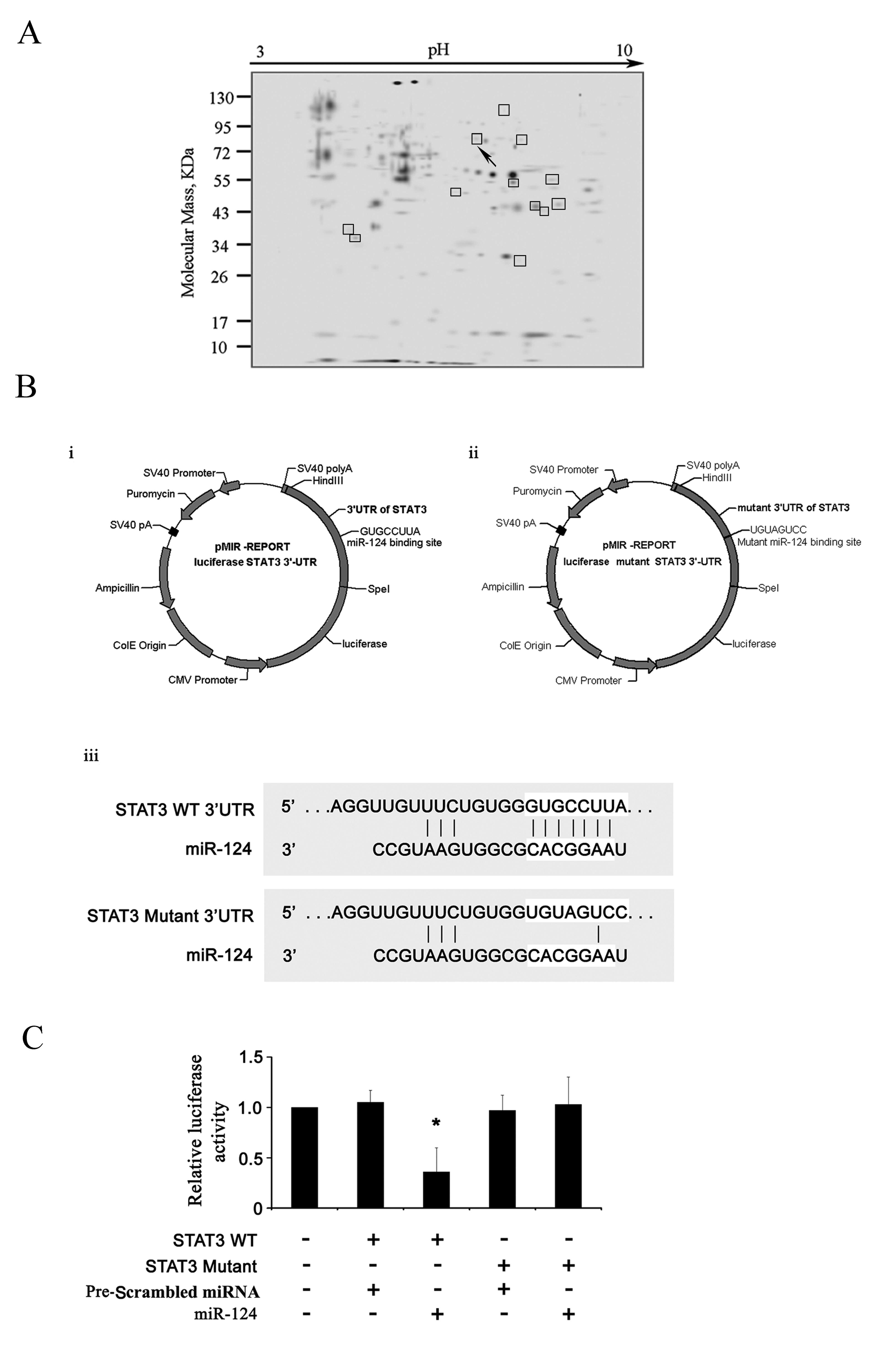

Supplement: Figure S1 — STAT3 is a direct target of miR-124 . (A) Two dimensional gel electrophoresis analysis on whole cell extracts from SW480 cells treated with Pre-miR-124 or Pre-Scrambled control miRNA. Proteins in the gel were stained with CBB (Coomassie brilliant blue) G-250. 12 protein spots circled are presumably down-regulated by miR-124. Arrowhead represents STAT3. (B) Design of luciferase reporter vectors containing a CMV promoter driving expression of a luciferase cDNA fused to the STAT3 3′UTR (B.i) or to the mutated STAT3 3′UTR (B.ii). The miR-124 WT binding site and mutated binding site in the 3′UTR of STAT3 are shown in B.iii. (C) MiR-124 binding site within STAT3 3′UTR mediates miR-124 control of STAT3 translation. SW480 cells were co-transfected with the luciferase constructs, Pre-miR-124 or Pre-Scrambled miRNA control, respectively. Cell lysate was collected and assayed for luciferase activities 48 h after transfection. Cells were transfected with a pMIR-REPORT miRNA expression reporter as control. Pre-miR-124 significantly decreases luciferase activity containing a WT miR-124 binding site but not a mutant binding site. (TIF) [file pone.0070300.s001.tif]
